# Editorial: Coinfections of Lyme disease and other tick-borne diseases

**DOI:** 10.3389/fmicb.2023.1140545

**Published:** 2023-02-09

**Authors:** Jie Feng, Tao Lin, Andrei D. Mihalca, Qingli Niu, Marinda C. Oosthuizen

**Affiliations:** ^1^School of Basic Medical Sciences, Lanzhou University, Lanzhou, China; ^2^State Key Laboratory of Veterinary Etiological Biology, College of Veterinary Medicine, Lanzhou University, Lanzhou, China; ^3^Alkek Center for Metagenomics and Microbiome Research, Baylor College of Medicine, Houston, TX, United States; ^4^University of Agricultural Sciences and Veterinary Medicine of Cluj-Napoca, Cluj-Napoca, Romania; ^5^State Key Laboratory of Veterinary Etiological Biology, Key Laboratory of Veterinary Parasitology of Gansu Province, Lanzhou Veterinary Research Institute, Chinese Academy of Agricultural Sciences, Lanzhou, Gansu, China; ^6^Department of Veterinary Tropical Diseases, Faculty of Veterinary Science, University of Pretoria, Pretoria, South Africa

**Keywords:** coinfections, tick-borne diseases, lyme disease, babesiosis, tick

Lyme borreliosis (LB), caused by *Borrelia burgdorferi* sensu lato (s.l.), is the most common tick-borne disease (TBD) in the northern hemisphere. Beyond *Borrelia*, ticks can transmit other pathogens, such as *Rickettsia, Babesia, Anaplasma, Ehrlichia*, viruses, etc. The last two decades have seen a sharp increase in TBDs with around 300,000 LB cases every year in the United States and 100,000 cases in Europe. Considering the low sensitivity of current diagnostics, we have only seen the “tip of the iceberg” with regard to the actual cases of TBDs. Ticks, deemed as the second most important arthropod vector of diseases after mosquitoes, often carry multiple species of pathogens and/or opportunistic pathogens, which can infect people simultaneously following the ticks' bite. In other words, it is called multi-species infections, or coinfections. With ticks able to transmit several pathogens in one bite, coinfections may be “the rule, not the exception.” Comorbid human infection with more than one tick-borne pathogen (TBP) is often detected worldwide. Coinfection is of particular human health importance and is getting increased attention due to the interaction of pathogen species within the host, which makes diagnosis and treatment more challenging. The *B. burgdorferi* s.l. infection can cause temporary human immunosuppression and has been documented to boost transmission of *Babesia microti*. Therefore, additional tick surveillance and awareness programs are required for early detection of the TBPs-risk to human health.

This Research Topic aims to present the prevalence of tick-borne coinfections and to provide a better understanding of how the pathogens interact within the host, as well as to develop better strategies in diagnoses and treatments for TBDs and other vector-borne diseases. In this Research Topic, four articles have been published that enrich our knowledge of the prevalence of tick-borne coinfections, tick-borne pathogens, and new diagnosis methods of LB.

Previously, the TBPs were investigated mostly in rural or remote areas without enough data from urban areas. Borşan et al. studied the distribution of TBPs in ticks and some wild animals in the city of Cluj-Napoca, Romania. Within *Ixodes ricinus*, the most prevalent human-biting tick species in Romania, *B. burgdorferi* s.l. displayed a detection rate of 36.6–37.9%, posing a great risk to human health. The other TBPs in *I. ricinus* consisted of *Anaplasma phagocytophilum* and two other rickettsia species. Besides, *Borrelia* spp. and *A. phagocytophilum* were detected in urban wildlife, such as rodents and birds. This study also displayed frequent occurrence of coinfections in both questing and engorged ticks, with the most commonly detected pathogens of *Borrelia* spp. and *Rickettsia* spp. in questing ticks as well as *Borrelia* spp. and *A. phagocytophilum* in engorged ticks, respectively. Coinfections with various TBPs were detected in 34.3% of questing *I. Ricinus* ticks and 69.2% of all engorged ticks. Moreover, coinfections with three pathogens were also identified though they are less common.

Many cases of tick-borne coinfections were reported in the United States and Europe. Yet, little is available about the coinfection prevalence of TBPs around the southern hemisphere. Chandra et al. analyzed the complete microbiome and live microbial species in female *Ixodes holocyclus* obtained from coastal areas of northeastern New South Wales, Australia. The combination of 16S rRNA gene sequencing and total RNA sequencing was adopted in this study to depict the endosymbiont profile within *I. holocyclus*, as well as other bacterial and viral organisms with unknown pathogenicity. *Candidatus* Midichloria spp., *Candidatus* Neoehrlichia arcana, and *Candidatus* Neoehrlichia australis were confirmed to be partial bacterial components of the core microbiota in *I. holocyclus*. Seven virus species were also detected, three of which were novel species never identified in *I. holocyclus*. Notably, one of the four previously identified virus species was considered to have pathogenic potential based on its phylogenetic relationship to other tick-associated pathogens. Detecting and evaluating potential pathogens represent a meaningful strategy to control emerging infectious diseases in the future. Meanwhile, this study provides an effective approach to investigating the diversity of tick microbiome and virome.

Due to the unique physiological characteristics of *B. burgdorferi* s.l., including varied antigenic epitopes and the slow growth rate, reliable and sensitive methods for detecting *B. burgdorferi* s.l. are still in shortage, bringing difficulty to the diagnosis of LB and its coinfections. Therefore, laboratory tests to determine and distinguish *B. burgdorferi* s.l. and other TBPs play a vital role in accurate diagnosis and consequent antibiotic treatment. Shan et al. developed a novel LB diagnosis assay by using internally controlled quantitative PCR (Ter-qPCR) to detect the terminase large subunit (*terL*) gene encoded by *Borrelia* prophages. The *terL* gene is species-specific and detected only in *B. burgdorferi* s.l.; moreover, the *terL* gene is a multicopy gene in *Borrelia* genome sequences, which largely increased the sensitivity of the assay. Novel nucleic acid-based detection methods are expected to complement current serological tests.

With regard to *B. burgdorferi* s.l.-related coinfections, *Babesia microti* is commonly involved, leading to the great severity of the disease. *Babesia* alone can cause a TBD called human babesiosis, with *B. microti* as the primary pathogen. Puri et al. summarized genomic structure, phylogeny, genetic variability, antigenic polymorphism, and human immune targets of *B. microti*, as well as its pathogenesis including acute infection, persistent infection, hemolytic anemia, and hypercoagulability. This integrative review on *B. microti* will benefit studies on the selection of new detecting biomarkers and drug-related genes, and guide the development of drugs and vaccines in the future.

TBDs involved with coinfections are still poorly understood due to the lack of thorough understanding of the pathogens themselves and interactions among ticks, pathogens, and the human host. To accurately diagnose and treat patients, it is vital to have a profound and systematic understanding of coinfections in the context of TBDs. The knowledge we have learned from tick-borne coinfections can also be applied to other vector-borne coinfections.

## Author contributions

JF, TL, AM, QN, and MO contributed to the conception of the editorial. JF wrote the manuscript. TL, AM, QN, and MO reviewed and revised the manuscript. All authors contributed to the article and approved the submitted version.

